# *Angelicae Pubescentis* Radix Remitted Intestine Damage in Mice Induced by *Escherichia coli* via Mediating Antioxidant Defense, Inflammatory Mediators, and Restoring Gut Microbiota

**DOI:** 10.3390/vetsci12040354

**Published:** 2025-04-10

**Authors:** Kehong Deng, Chang Xu, Qing He, Muhammad Safdar, Mudassar Nazar, Xiaocong Li, Kun Li

**Affiliations:** 1 Department of Pharmacy, Hubei Three Gorges Vocational and Technical College, Yichang 443000, China; yc-dkh@163.com; 2 College of Veterinary Medicine, Nanjing Agricultural University, Nanjing 210095, China; 9201710122@stu.njau.edu.cn (C.X.); 9201710121@stu.njau.edu.cn (Q.H.); 3 Department of Breeding and Genetics, Cholistan University of Veterinary and Animal Sciences, Bahawalpur 63100, Pakistan; msafdar@cuvas.edu.pk; 4 Department of Clinical Medicine and Surgery, Faculty of Veterinary Sciences, Constituent College Burewala, University of Agriculture Faisalabad, Burewala 61010, Pakistan; mudassar786.rana@gmail.com

**Keywords:** *Angelicae Pubescentis* Radix, bacterial diarrhea, intestinal micro-ecosystem, antioxidant defense, gut microbiota

## Abstract

This study aims to explore the therapeutic potential of *Angelicae Pubescentis* Radix (APR), a traditional Chinese medicine that is widely known for its anti-inflammatory, anti-oxidative, and anti-microbial properties, using a mouse model. In this study, 30 mice were selected and divided into three groups: control group (CD), infection group (ED), and treatment group (TD). Mice in the TD were gavaged with APR oil (0.15 mL/kg/day) for 20 days, while mice in the CD and ED received an equal volume of normal saline. On the 21st day, mice in the ED and TD were infected with multi-drug-resistant *E. coli* (1 × 10^7^ CFU/mL) derived from diarrheal yak. Twenty-four hours later, all mice were euthanized, and blood, organs, and intestinal samples were collected for analysis. The results of intestinal sections and intestinal bacterial load revealed that APR treatment significantly reduced (*p* < 0.05) both bacterial load and intestinal injury. Serum analysis indicated that APR treatment also alleviated the inflammation and oxidative stress induced by *E. coli* infection. Intestinal microbiota sequencing further showed that APR treatment increased the abundance of intestinal probiotics such as *Ligilactobacillus*, *Paludicola*, and *Blautia_A_1417806* while also enhancing the enrichment of functional pathways associated with antioxidant defense. In conclusion, APR treatment effectively alleviates diseases caused by *E. coli* infection, promotes the growth of beneficial intestinal bacteria, and improves the antioxidant capacity in animals. Additionally, these findings confirm APR’s role in addressing immediate effects rather than chronic adaptations. Future studies should investigate the prolonged effects of APR treatment beyond the acute phase.

## Simple Summary

This study investigated the therapeutic potential of *Angelicae Pubescentis* Radix (APR) in mice infected with *Escherichia coli* (*E. coli*) and found that APR treatment significantly reduced both bacterial load and intestinal injury. Serum analysis indicated that APR treatment also alleviated the inflammation and oxidative stress induced by *E. coli* infection. Intestinal microbiota sequencing further showed that APR treatment increased the abundance of intestinal probiotics such as *Ligilactobacillus*, *Paludicola*, and *Blautia_A_1417806* while also enhancing the enrichment of functional pathways associated with antioxidant defense.

## 1. Introduction

Bacterial diarrhea, primarily caused by enteropathogenic bacteria such as *E. coli*, *Salmonella* [1], and *Shigella* [1], is a major public health issue worldwide. These pathogens typically enter the human body via contaminated food or water [2], disturbing the intestinal microecological balance [3], triggering an inflammatory response [4], and resulting in symptoms such as diarrhea, abdominal pain, fever, and others. Traditional treatments largely rely on antibiotics to rapidly control the infection by killing or inhibiting the growth of bacteria [5]. However, the overuse and misuse of antibiotics have led to the emergence of drug-resistant “superbugs” [6] and can disrupt the natural intestinal microbiota, leading to secondary infections or chronic intestinal diseases [7], such as *Clostridium difficile*-associated diarrhea (CDAD), which complicates treatment and increases risks [8]. In this regard, traditional Chinese medicine (TCM) has gained attention as a promising alternative due to its natural, safe, and relatively inexpensive characteristics [9,10,11,12]. Many studies have shown that compound Chinese herbal extracts can significantly improve immune functions [13], maintain the balance of intestinal flora [14], and enhance antioxidant defense, thereby reducing disease incidence [15,16]. However, the mechanisms of action of TCM and antibiotics differ, and with increasing bacterial resistance, blindly replacing antibiotics with Chinese medicine may not achieve the desired effects.

*Angelicae Pubescentis* Radix (APR), a member of the Umbelliferae family, is rich in volatile oils, coumarins, polysaccharides, and other bioactive compounds [17]. These compounds demonstrate significant immune-regulating, anti-inflammatory, and antioxidant effects [18]. Specifically, coumarin compounds, such as imperatorin, possess remarkable anti-inflammatory effects [19] by inhibiting the nuclear factor kappa B (NF-κB) signaling pathway, thereby alleviating intestinal inflammatory mediators and promoting intestinal mucosal repair [20]. Additionally, polysaccharides in APR can activate immune cells and enhance the body’s ability to resist bacterial invasion [17,20]. Similarly, coumarins like columbianadin (CBN) and osthole also inhibit NF-κB activation, reducing the expression of pro-inflammatory cytokines like tumor necrosis factor-α (TNF-α), interleukin-6 (IL-6), and interleukin-1β (IL-1β) [17,20]. The suppression of Nucleotide-binding oligomerization domain-containing protein 1 (NOD1)/NF-κB signaling to block LPS-induced cytokine production in immune cells has been proved [20]. CBN inhibits Janus Kinase 2/Signal Transducer and Activator of Transcription 3 (JAK2/STAT3) phosphorylation, interrupting cytokine-derived inflammatory cascades [17]. Additionally, coumarins modulate Signal Transducer and Activator of Transcription (MAPK) pathways (e.g., p. 38), reducing oxidative stress and tissue damage [17,20]. Hence, coumarins like osthole and CBN enhance intestinal barrier function by reducing oxidative stress and inflammatory mediators’ release [20]. Additionally, Bergapten and umbelliferone exhibit analgesic and anti-inflammatory effects, potentially stabilizing gut epithelial cells during immune challenges [20].

APR polysaccharides bind to Toll-like receptors (TLRs) and pattern recognition receptors (PRRs), activating macrophages and dendritic cells [21,22]. This triggers the production of cytokines, e.g., Granulocyte-Macrophage Colony-Stimulating Factor (GM-CSF), that promote hematopoiesis and immune cell differentiation, increased phagocytic activity, and antigen presentation, boosting adaptive immune response [21,22]. *Angelica sinensis* polysaccharides (ASP) activate the Wingless-related integration site/β-catenin pathway (Wnt/β-catenin), reducing oxidative injury in gut epithelial cells [23]. Similarly, ASP-Hb-AP (a polysaccharide variant) upregulates antioxidant enzyme expression, e.g., superoxide dismutase (SOD) and catalase (CAT), in intestinal cells, alleviating oxidative damage. Polysaccharides bind TLRs on epithelial cells, inducing anti-inflammatory cytokine release [21] and also stimulating mucin (MUC2) [23] production via gut microbiota, strengthening the mucosal layer [23].

The gut, as the largest digestive organ in the body, plays a crucial role in overall health. The intestinal microbiota is closely associated with various physiological processes, including metabolism, immunity, and nutrient absorption [24]. The intestinal flora, comprising the microbiota, its metabolites, and the environment in which they exist, is fundamental to maintaining body health [25]. Once disrupted, it can lead to a range of diseases [26]. At present, over 1000 species of intestinal microbial members have been identified in the human gut [27]. Under normal conditions, the intestinal microbiota remains in a stable state, but an abnormal physiological condition can trigger a variety of health issues [28]. Additionally, this intestinal microbiota provides essential nutrients and supports the normal functioning of the intestinal barrier, playing a key role in defending against pathogens [29,30,31]. The interaction between symbiotic bacteria, metabolites, intestinal epithelial cells, and the immune system helps to maintain the balance of the intestinal environment and serves as a natural barrier against pathogen invasion [32]. Therefore, in this study, *E. coli* was employed to induce bacterial diarrhea in model animals, and the therapeutic effects of APR were investigated from the perspectives of intestinal microecological changes, antioxidant stress, and inflammation, as well as the potential of replacing antibiotics with traditional Chinese medicine.

## 2. Materials and Methods

### 2.1. Oil Extraction

*Angelicae Pubescentis* Radix (1.0 kg) was purchased from a Chinese herb farm in Yichang, China. The main roots were carefully collected and shade-dried. Then, the velamina of the roots were removed, and the roots were soaked in hexyl hydride (5 L) for 10 h. The resulting extracts were concentrated at 45 °C and dried via vacuum to achieve oil with a concentration of (2 g/mL) as depicted in a previous study [33]. The key active ingredients in the APR oil extract include coumarins such as imperatorin, CBN, osthole, Bergapten, and umbelliferone, along with volatile oils and APR polysaccharides [17,20].

### 2.2. Animal Experiment

Thirty ICR mice (15 male and 15 female), four weeks old and weighing 24.16 ± 0.75 g, were obtained from the Experimental Animal Center at Yangzhou University. Mice were acclimatized for 3 days under controlled conditions (26 °C, 50–60% humidity, 12 h light/dark cycle). Feed and water were provided ad libitum, with environmental parameters monitored daily. Four-week-old ICR mice were selected to standardize immune and metabolic responses, as juvenile rodents are widely used in enteric infection models to ensure uniformity in pathogen susceptibility and therapeutic outcomes [3,15]. This age minimizes developmental variability while allowing acute observation of *E. coli*-induced intestinal damage and APR intervention. The feed used in the given study was purchased from Yizheng Anliu Biotechnology Co. Ltd. (Yizheng, China; standards: GB14924.1-2010, GB14924.2-2010, GB14924.3-2010) and mainly consisted of corn, wheat, soybean meal, bran, fish meal, various amino acids, and multivitamins. The feed provided was composed of ≤10% moisture, ≥19% protein, ≥4% crude protein, ≤5% crude fiber, ≤8% crude ash, 1.0–1.8% calcium, 0.6–1.2% phosphorus, with calcium to phosphorus ratio (Ca: P); 1.2:1–1.7:1, ≥0.92% lysine, and methionine + cystine ≥ 0.53%. The remaining ~48% was carbohydrate (corn) content. The feed was stored in dry, ventilated conditions at a temperature of 20–25 °C, with a relative humidity of 50–60%, in a sealed (air-tight) pack to avoid environmental exposure. The shelf life of feed was 3 months (2 months in the rainy season).

**Figure 1:**
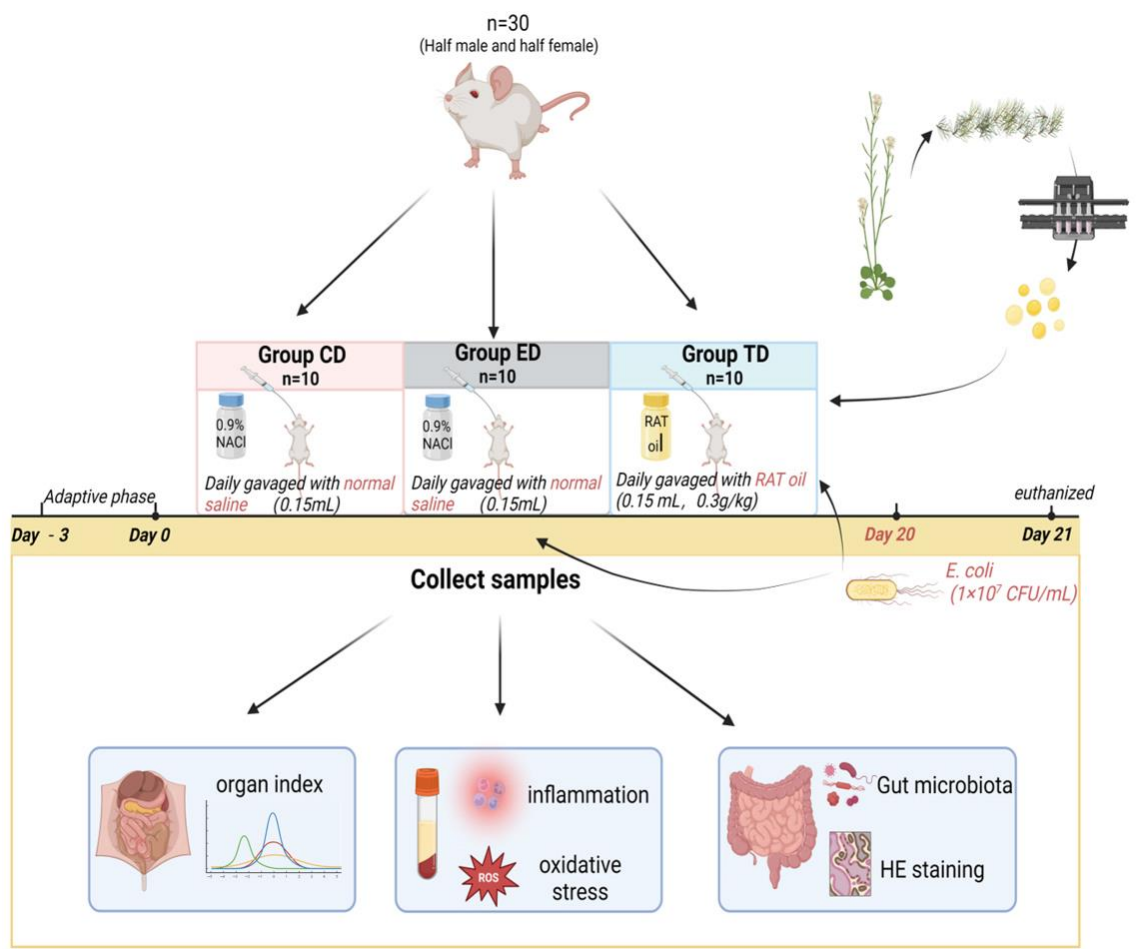
Experimental design of the mice model induced by *E. coli*.

After three days of acclimatization, the mice were randomly divided into three groups: the control group (CD), the infection group (ED), and the treatment group (TD). ICR mice in the TD were gavaged with APR oil (0.15 mL/kg/day) for 20 days, while those in the CD and ED received an equal volume of normal saline. On the 21st day, the ED and TD were inoculated with multi-drug-resistant *E. coli* (clinical isolate PP859186, 1 × 10⁷ CFU/mL) derived from diarrheal yak [3]. The strain’s pathogenicity is attributed to its clinical origin and resistance profile, though serotype-specific markers (e.g., K88/K99) were not characterized. Meanwhile, mice in the CD were administered an equivalent amount of Luria–Bertani nutrient medium (Solarbio Science & Technology Co., Ltd., Beijing, China). Twenty-four hours post-inoculation, all mice were euthanized to collect blood, organs, and intestinal samples. The key parameters, such as body weight, diarrhea rate and mortality, organ index, organ bacterial loads, serum oxidation resistance, inflammation levels, and microbiota composition, were analyzed (Figure 1).

### 2.3. Pathological Analysis

Jejunum tissues (0.5–1 cm/animal) from mice in CD, TD, and ED were collected and fixed in a 4% formaldehyde solution for 48 h. The fixed samples were then sent to Wuhan Pinuofei Biological Technology (China) for hematoxylin and eosin (H&E) staining. Pathological analysis of villus integrity and damage was conducted using an Olympus CX33 microscope (Olympus, Hachioji, Tokyo, Japan), as described in a previous study [15].

### 2.4. Examination of Antioxidant and Inflammatory Levels in Mice

Blood samples were collected and centrifuged at 3500× *g* for 20 min to separate the serum. The obtained serum samples were analyzed for antioxidant and inflammatory markers using commercial assay kits, including malondialdehyde (MDA), SOD, total antioxidant capacity (T-AOC), glutathione peroxidase (GSH-Px), IL-6, IL-10, TNF-α and IL-1β, sourced from Solarbio Science & Technology Co, Ltd. (Beijing, China) and Jiancheng Bioengineering Research Institute (Nanjing, China).

### 2.5. Microbiota Sequencing and Analysis

The total microbial DNA was extracted from rectal samples of mice in the CD (*n* = 5), TD (*n* = 5), and ED (*n* = 5) groups using the FastDNA Spin Kit for Feces (Beijing Think-Far Technology Co, Ltd., Beijing, China). The quality and quantity of DNA were checked via Ultramicrospectrophotometer YSNano-100 (Yeasen Biotechnology, Shanghai, China) and agarose gel electrophoresis (1.5%), following the protocol described in a previous study [34,35]. The V3-V4 region of the 16S rRNA gene was amplified using 338F-806R primer pairs (F: 5′-ACTCCTACGGGAGGCAGCAG-3′, R:5′-GGACTACHVGGGTWTCTAAT-3′) [36]. The amplified products were used to construct sequencing libraries via the Hieff NGS^®^ OnePot II DNA Library Prep Kit for Illumina^®^ (Yeasen, China). Microbiota sequencing was subsequently performed on the Illumina platform at Bioyi Biotechnology Co, Ltd. (Wuhan, China).

The raw sequencing data from the CD, TD, and ED underwent quality control and denoising via Divisive Amplicon Denoising Algorithm 2 (DADA2, Version: 1.26.0) [37]. The filtered data were used to produce amplicon sequence variants (ASVs) by blasting with the Greengenes database with ≥97% similarity and assigned taxonomy table via Quantitative Insights into Microbial Ecology (QIIME 2 Version: 2024.10) platform [38]. A Venn diagram was generated to visualize shared ASVs among the CD, TD, and ED [39]. The analysis of alpha and beta diversities of mice in different groups was conducted via the QIIME2 platform to explore the species diversity of samples and groups [40]. Species difference and biomarker analyses were performed via Linear discriminant effect Size (LefSe) analysis and analysis of variance (ANOVA) [41]. Finally, microbiota function prediction was performed using the Phylogenetic Investigation of Communities by Reconstruction of Unobserved States (PICRUSt2; Version: 2.6.) [42], and the abundance of metabolic pathways among different groups was analyzed by employing the Kyoto Encyclopedia of Genes and Genomes (KEGG) and MetaCyc Metabolic Pathway (MetaCyc) databases [43].

### 2.6. Statistical Analysis

Variances among different mice groups (CD, TD, and ED) were analyzed using ANOVA and Student’s *t*-test via IBM SPSS (25.0). One-way ANOVA with Tukey’s post hoc test was used for comparisons across all three groups (e.g., body weight changes, organ indices, bacterial loads across intestinal segments, antioxidant markers, inflammatory cytokines, alpha diversity indices, and taxonomic and functional pathway abundances), while Student’s *t*-test was applied for pairwise group comparisons (e.g., weight loss in ED vs. CD/TD, bacterial load reduction in TD vs. ED, recovery of antioxidant markers in TD vs. ED). Results are presented as means ± standard error of the mean (SEM), with statistical significance set at *p* < 0.05.

## 3. Results

### 3.1. Effect of APR Oil on Body Weight in E. coli-Induced Mice

**Figure 2:**
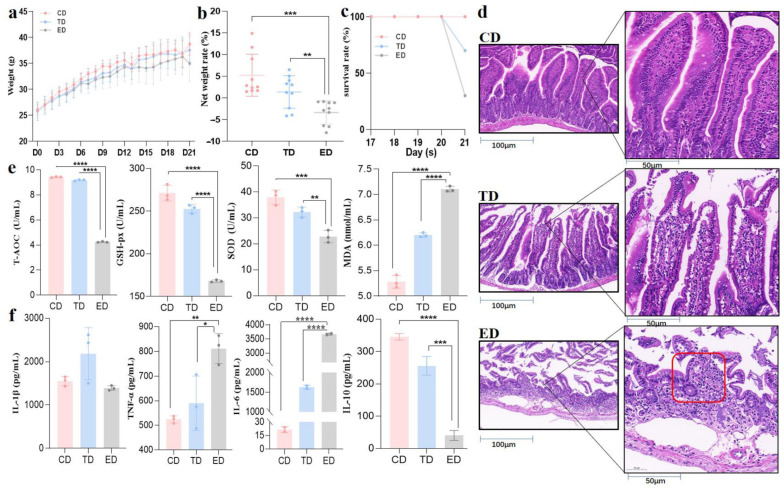
APR oil regulated body weight, intestinal integrity, inflammation, and antioxidant capacity in *E. coli*-induced mice. (**a**) Body weight, (**b**) Net weight change on day 21, (**c**) Survival rate, (**d**) H&E staining (red circled area in group ED shows severely damaged and atrophied villi, (**e**) Antioxidant capacity, (**f**) Inflammation levels. Scale bar = 50 μm. Statistical significance is indicated as * *p* < 0.05, ** *p* < 0.01, *** *p* < 0.001, **** *p* < 0.0001. Data are presented as mean ± SEM.

Following *E. coli* induction, no significant differences (*p* ≥ 0.05) in body weight were observed among the groups (Figure 2a). However, post-infection, the ED displayed a net weight loss (~3%), while the TD and CD experienced weight gain. By the experiment’s conclusion, the CD exhibited the most substantial relative weight gain, averaging approximately 10%, while the TD showed a moderate increase of ~5%. In contrast, the ED demonstrated a significant (*p* < 0.05) average weight loss of ~3% (Figure 2b). These findings highlight distinct weight trends among the groups, reflecting the physiological impacts of infection and treatment. Additionally, the CD maintained a 100% survival rate throughout the experiment, while the TD had a slight reduction in survival, and the ED experienced a significant drop (*p* < 0.05) in survival rate by day 21 (Figure 2c).

The pathological examination (Figure 2d) of jejunum tissues revealed clear differences in the intestinal tissue integrity. The CD exhibited intact intestinal mucosa, a well-organized tissue structure, and complete, neatly arranged villi. In contrast, the ED showed severe intestinal damage, including inflammatory cell infiltration, disrupted tissue structure, and villi characterized by obvious shortening, aggregation, and destruction. After treatment with APR oil, the infected mice maintained relatively complete intestinal mucosa and villi, although there were some defects in the upper mucosa and other phenomena. The integrity was markedly higher compared to the ED, and the structure was close to normal.

Serum oxidative stress markers also highlighted the protective effects of APR oil. T-AOC, GSH-px, and SOD levels, which were significantly decreased (*p* < 0.05) in the ED, showed recovery in the TD, approaching levels observed in the CD. Conversely, MDA levels, which were elevated significantly (*p* < 0.05) in the ED, were slightly alleviated in the APR oil treatment group (Figure 2e). Inflammatory cytokine analysis further supported these findings. IL-6 and TNF-α levels were significantly elevated (*p* < 0.05) in the serum of the ED but returned to normal in the TD. Additionally, IL-10, an anti-inflammatory cytokine, was substantially higher in the TD compared to the ED, which exhibited minimal levels (Figure 2f). Therefore, these results suggest that APR oil effectively mitigates weight loss, restores intestinal integrity, and alleviates inflammation and oxidative stress in *E. coli*-induced mice.

### 3.2. Effect of APR Oil on Organ Index and Bacterial Load in E. coli-Induced Mice

After blood collection from the eyeballs, about 0.1 g of heart, liver, spleen, lung, kidney, duodenum, jejunum, ileum, cecum, and colon were weighed and placed in a sterilized 2 mL centrifuge tube, and 1 mL of normal saline and two grinding beads were added. After grinding, the supernatant was taken and diluted 10 times its original volume. The diluted 20 μL bacterial solution was inoculated into the MacConkey agar and cultured in the incubator at 37 °C for 18 h. The colony number was recorded, and the bacterial load in each organ and intestine was calculated.

**Figure 3:**
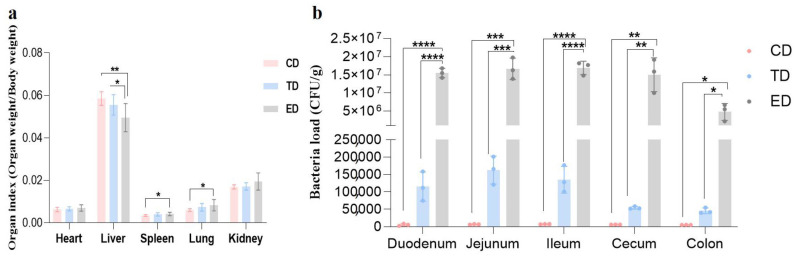
Organ index and bacterial loads in different intestinal segments of mice in different groups. (**a**) Organ index, (**b**) Bacterial loads. Statistical significance is indicated as * *p* < 0.05, ** *p* < 0.01, *** *p* < 0.001, **** *p* < 0.0001. Data are presented as mean ± SEM.

The organ indices of the spleen, liver, and lungs in the ED were significantly different (*p* < 0.05) from those in the control group, indicating notable organ changes due to *E. coli* infection. In contrast, the organ indices in the APR oil treatment group were similar to those observed in the CD, suggesting that APR oil may help mitigate these changes. The bacterial load in the intestinal tract also displayed interesting trends. In the ED, the bacterial load in the duodenum, jejunum, ileum, cecum, and colon were significantly increased (*p* < 0.05) compared to the CD and TD. Specifically, the bacterial load in the ED was approximately 100 times higher than that in the TD, indicating a substantial increase in intestinal bacteria due to *E. coli* infection (Figure 3a,b).

### 3.3. Effect of APR Oil on Intestinal Flora Composition in Mice Induced by E. coli

Sequencing data revealed more than 72,000, 64,000, and 62,000 unfiltered original sequences from the CD, ED, and TD, respectively. After denoising and filtering, at least 51,000, 42,000, and 41,000 high-quality sequences were obtained from these groups (Table 1).

**Figure 4:**
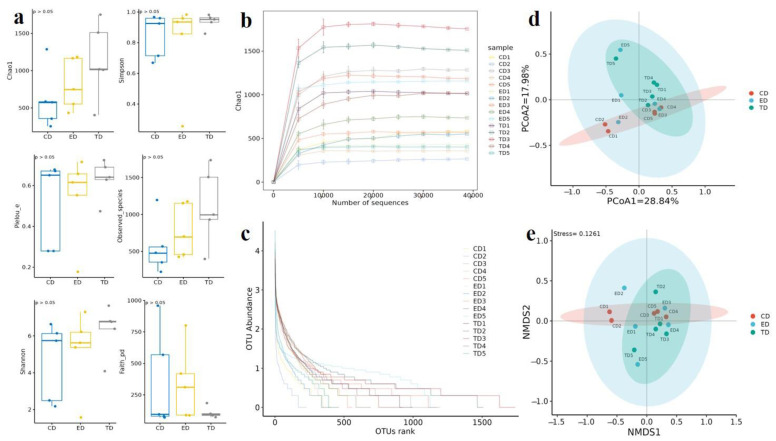
Alpha and beta diversity analysis. (**a**) Diversity indexes, (**b**) Rarefaction curve, (**c**) Rank abundance curve, (**d**) Principal coordinate analysis (PCoA), (**e**) Non-metric multidimensional scaling (NMDS).

Alpha diversity analysis was employed to compare the microbial diversity within each group (Figure 4a). The Good’s coverage index ranged from 99.43 to 99.96% across the three groups, indicating that the sequencing results could reflect the entire bacterial structure. The α-diversity correlation index, a measure of species richness, was highest in the TD (1139.61 ± 406.73), followed by the ED (814.89 ± 367.2), and lowest in the CD (611.41 ± 679.61).

The Shannon index, which reflects both richness and evenness, was also highest in the TD (6.34 ± 2.24), followed by the ED (5.21 ± 3.65) and the CD (4.64 ± 2.18). The Pielou_e index, which measures evenness, ranged from 0.278 to 0.678 in the CD, 0.177–0.714 in the ED, and 0.474–0.724 in the TD. The Observed_species index, representing the number of distinct species observed, ranged from 288.1 to 1202.5 in the CD, 421.7–1174.2 in the ED, and 403.2–1734 in the TD (Table 2). Additionally, both the rarefaction curve (Figure 4b) and the rank-abundance curve (Figure 4c) approached a plateau with an increasing number of sequences, suggesting that the sequencing depth was sufficient to reflect the complete microbiota composition and maximum species diversity has been captured. However, some curves (e.g., CD1 and ED1) appear to continue increasing slightly, suggesting that additional sequencing might uncover more diversity in these samples. Overall, sequencing depth seems sufficient for robust analyses, but further sequencing could be considered for samples where saturation is not fully achieved.

β-diversity analysis, including PCoA (Figure 4d) and NMDS (Figure 4e), revealed significant differences (*p* < 0.05) in microflora composition among the three groups, with dispersed sample distributions indicating group-specific microbial variations.

### 3.4. Effect of APR Oil on Intestinal Flora Composition in E. coli-Induced Mice

At the phylum level, 56 phyla were identified across the groups. Bacteroidota (CD: 28.47%, ED: 29.17%, TD: 47.29%) and Firmicutes_D (CD: 25.13%, ED: 32.93%, TD: 11.59%) were the most abundant. Campylobacterota was most abundant in the CD (32.97%), Proteobacteria dominated the ED (14.00%), and Firmicutes_A was prevalent in the TD (19.21%). Other less abundant phyla included Deferribacterota (CD: 5.72%, ED: 1.21%, TD: 0.54%), Actinobacteriota (CD: 0.48%, ED: 3.08%, TD: 1.29%), and Desulfobacterota_I (CD: 0.18%, ED: 0.46%, TD: 1.65%).

At the class level, Bacteroidia (CD: 28.47%, ED: 29.12%, TD: 47.27%) and Bacilli (CD: 25.13%, ED: 32.93%, TD: 11.59%) were the most prevalent. Campylobacteria, abundant in the CD (32.97%), was reduced in the ED (5.28%) and TD (7.27%) groups. Gammaproteobacteria was most abundant in the ED (12.85%), with lower levels in CD (0.52%) and TD (9.65%). Clostridia_258483 (CD: 4.17%, ED: 7.98%, TD: 19.20%) was present in all groups. In total, 9176 ASVs were generated, with 1880 unique to the CD, 2872 unique to the ED, and 4424 unique to the TD.

**Figure 5:**
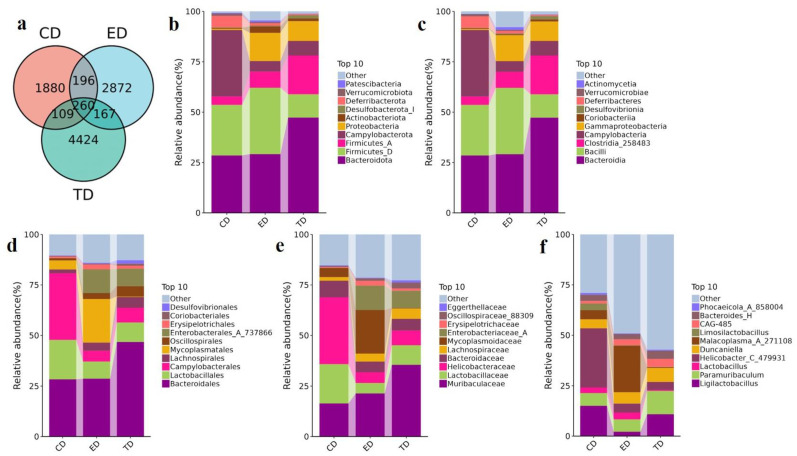
Venn diagram of ASVs and APR-mediated changes in the intestinal microbiota of *E. coli*-induced mice in different taxa. (**a**) Venn diagram of ASVs, (**b**) Phylum, (**c**) Class, (**d**) Order, (**e**) Family, (**f**) Genus.

At the genus level, *Ligilactobacillus* was most abundant in the CD and TD (CD: 15.05%, TD: 10.86%) but scarce in the ED (2.18%). *Helicobacter_C_479931* (29.34%) and *Paramuribaculum* (6.29%) were predominant in the CD, while *Paramuribaculum* (11.59%) and *Helicobacter_D* (8.74%) were more common in the TD. *Malacoplasma_A_271108* (22.97%) was the most abundant genus in the ED. Additionally, 260 ASVs were shared across all three groups (Figure 5a). Figure 5b–f shows the top ten phyla, classes, orders, families, and genera among all three groups.

**Figure 6:**
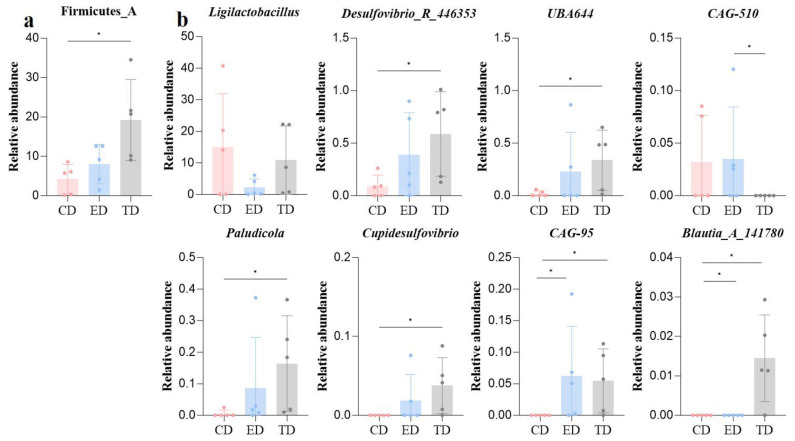
Microbiota differences among mice in different groups revealed by ANOVA analysis (**a**) Phyla and (**b**) Genus. Statistical significance is indicated as * *p* < 0.05, Data are presented as mean ± SEM (n = 5).

Then, the re-analysis demonstrated significant differences (*p* < 0.05) in both phylum and genus composition. Firmicutes A was notably higher in the TD (Figure 6a), while *Ligilactobacillus* was more abundant in the CD and TD. The TD also showed increased levels of *Desulfovibrio R 446353*, *UBA644*, *Paludicola*, *Cupidesulfovibrio*, and *Blautia_A_141780*, while *CAG-95* and *CAG-510* were more abundant in the ED (Figure 6b).

### 3.5. Analysis of Differential Metabolic Pathways Using KEGG and MetaCyc Databases

Differential metabolic pathways were analyzed using the KEGG and MetaCyc databases. The KEGG results revealed several pathways with LDA scores greater than 2 in the CD, including aminoacyl-tRNA biosynthesis, D-glutamine and D-glutamate metabolism, D-alanine metabolism, photosynthesis, lysine biosynthesis, ribosome production, protein export, mismatch repair, and peptidoglycan biosynthesis. In contrast, the ED showed higher LDA scores in pathways such as the biosynthesis of ansamycins, African trypanosomiasis, and the degradation of valine, leucine, and isoleucine.

**Figure 7:**
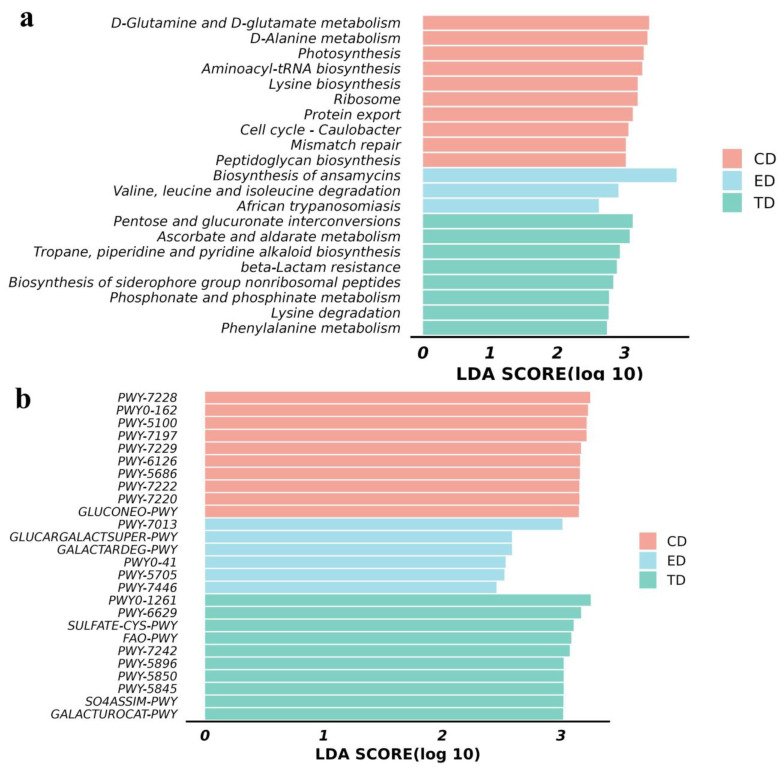
Functional analysis of mice microbiota (**a**) KEGG pathway analysis and (**b**) MetaCyc pathway analysis.

At LDA SCORE (log 10) in the CD, D-glutamine and D-glutamate metabolism, D-alanine metabolism, aminoacyl-tRNA biosynthesis, lysine biosynthesis in group ED; biosynthesis of ansamycins, valine, and leucine and isoleucine degradation, in group TD; pentose and glucuronate interconversions, ascorbate and aldarate metabolism, tropane, piperidine, and pyridine alkaloid biosynthesis, beta-lactam resistance, etc., related pathways were found to be significantly (*p* < 0.05) upregulated (Figure 7a). Similarly, MetaCyc pathway analysis indicated that in group CD, PWY-7228, PWY0-162, PWY-5100, PWY-7197, PWY-7229, in group ED; PWY-7013, glucargalactsuper-PWY, galactardeg-PWY, in group TD; PWY0-1261, PWY-6629, sulfate-CYS-PWY, FAO-PWY and PWY-7242 pathways were found to be significant (*p* < 0.05) expressed (Figure 7b). These findings highlight distinct metabolic shifts across the groups in response to *E. coli* infection and APR oil treatment (Figure 7a,b).

## 4. Discussion

Our findings provide strong evidence that APR exerts protective effects in the early stages of bacterial infection by reducing oxidative stress and modulating inflammatory responses. The significant reduction in bacterial load and improvement in antioxidant markers highlight the acute therapeutic potential of APR rather than suggesting prolonged microbiota shifts. While previous studies have explored the chronic benefits of herbal treatments, our study underscores the importance of early intervention [44]. During the experimental period, the body weight of mice in the CD showed a fluctuating upward trend. In contrast, the weight of mice in the TD and ED decreased initially after being exposed to *E. coli*, though the TD exhibited a slight recovery. This suggests that APR treatment may enhance resistance to bacterial infection, reducing symptoms such as diarrhea and weight loss [17].

Additionally, cytokine analysis revealed that IL-6 and TNF-α levels were elevated in the infection group but significantly reduced in the APR treatment group within 24 h, emphasizing the immediate anti-inflammatory effects of APR. This aligned with the study’s objective of assessing acute responses rather than inferring long-term immune modulation. IL-10 is a key anti-inflammatory cytokine that blocks immune cell activation and cytokine production in innate immune cell types [45]. Conversely, pro-inflammatory cytokines such as IL-1β and TNF-α are secreted in significant quantities during pathological conditions [46,47]. The experimental results showed that APR treatment significantly reduced the inflammatory response in mice post-*E. coli* challenge. The literature indicated that other Chinese herbs, like Licorice root, have also shown anti-inflammatory and soothing effects on the gastrointestinal tract in treating ulcers [48] and promoting healing by increasing mucus production in the stomach [48,49].

In the antioxidant defense system, T-AOC serves as an important measure of the antioxidant capacity of bioactive substances [50]. The enzymes such as SOD and GSH-Px work together to convert reactive oxygen species (ROS) into harmless substances, thereby protecting cells from oxidative damage [51]. Additionally, MDA content is an important parameter reflecting the antioxidant capacity of the body, which can reflect the rate and intensity of lipid peroxidation in the body and also indirectly reflect the degree of tissue peroxidation damage [52]. The T-AOC in the ED was significantly lower than that in the control group and APR treatment group (TD). All antioxidant indexes of TD mice showed that APR treatment could significantly enhance the ability of mice to resist oxidative stress and clear free radicals; this is consistent with previous results using APR to treat rheumatoid arthritis [18]. Furthermore, in the given study, it was observed that the intestinal bacterial load in the APR treatment group decreased by about 100 times compared with that in the ED. This is consistent with the results of intestinal biopsies, and APR can alleviate intestinal damage caused by *E. coli* [48].

From the alterations in intestinal microecology, Bacteroidota and Firmicutes_D are the common phyla with high abundance among the three groups. Nevertheless, the abundance of Firmicutes_A in the TD was significantly elevated. Firmicutes, mostly Gram-positive bacteria, play a crucial role in host nutrition and metabolism through the synthesis of short-chain fatty acids (SCFAs) [53]. At the genus level, it was found that eight genera had abundance differences. The abundance of *Ligilactobacillus*, *UBA644*, *Paludicola*, *Cupidesulfovibrio*, and *Blautia_A_1417806* had all increased in the APR treatment group. Among these, *Ligilactobacillus* is a well-established probiotic that has been shown to have antibacterial and anti-inflammatory properties [54,55] and has been used to treat or relieve diarrhea, colitis, mastitis, and other conditions [56,57]. *Paludicola* has rarely been reported in recent years, but it has been confirmed that it can produce propionate [58]. As a short-chain fatty acid, propionate has anti-inflammatory and immune homeostasis regulation functions [59] and can maintain the stability and health of intestinal microecology [60]. Similarly, *Blautia_A_1417806* is thought to have potential probiotic properties [61], having previously been found to help humans adapt to hypoxic environments [62], and it has also been found to produce butyrate, which is beneficial for gut health [63].

Similar results have been observed with berberin, which modulated gut microbiome by reducing the harmful bacteria and enhancing probiotic bacteria, thus increasing intestinal barrier function and metabolic health [64,65]. Berberine acts by directly reducing bacterial growth and modulating inflammatory pathways. Its effects on gut microbiota are well-documented, showing a significant increase in beneficial bacteria like *Lactobacillus* and *Bifidobacterium* [64,65]. These findings suggest that APR treatment enhances the abundance of beneficial gut microbiota, particularly SCFA-producing bacteria, which likely play a key role in alleviating inflammation and repairing intestinal damage caused by bacterial diarrhea, comparable to established results [64,65].

In terms of functional pathways, several significant differences were observed in the TD, including pathways related to pentose and glucuronate interconversions. Ascorbate and aldarate metabolism, tropane, piperidine, and pyridine alkaloid biosynthesis beta-lactam resistance, etc. Ascorbate metabolism, in particular, was linked to increased activity in pentose and glucuronate interconversions and D-galacturonate degradation, pathways that are known to enhance ascorbate synthesis [66]. Ascorbate plays a vital role in promoting iron absorption, boosting immunity, preventing scurvy, and acting as a potent antioxidant by binding ROS and converting to monodehydroascorbic acid to mitigate oxidative stress [67]. Additionally, pathways related to vitamin K synthesis, such as menaquinol biosynthesis, showed increased expression. The reduced form of vitamin K (vitamin K-hydroquinone, VKH2) has been identified as an antioxidant that can effectively inhibit ferroptosis [68]. These findings suggest that APR treatment enhances the expression of antioxidant functional pathways, thereby alleviating oxidative stress in mice. These findings suggest that APR treatment can alleviate oxidative stress in mice by increasing the expression of functional pathways in antioxidants [69].

While APR exhibited acute therapeutic efficacy against *E. coli*-induced intestinal injury, its clinical translation needs to address its stability, bioavailability, and regulatory measures. Coumarins and polysaccharides as bioactive compounds from APR require technical formulation strategies, e.g., nanoencapsulation and enteric coatings, to increase their stability and absorption [17,28]. Regulatory pathways need rigorous standardization of extracts, lengthy safety assessments, and adherence to good management practices (GMPs) protocols, as exemplified by successful TCM-derived therapeutics like artemisinin [9,10]. Future research should prioritize pharmacokinetic studies and collaborative regulatory frameworks to bridge traditional use with modern therapeutic validation.

## 5. Conclusions

This study showed that APR effectively neutralizes the acute intestinal damage caused by *E. coli* infection in mice. APR treatment significantly reduced bacterial load and alleviated oxidative burden by T-AOC, SOD, GSH-Px, and downregulated pro-inflammatory cytokines, e.g., IL-6, TNF-α, while upregulated the anti-inflammatory IL-10. Histopathological results confirmed APR’s role in preserving intestinal mucosal integrity and villus structure. Furthermore, APR enriched the beneficial genera such as *Ligilactobacillus*, *Paludicola*, and *Blautia_A_1417806*, which are linked to SCFA production and antioxidant pathway activation. These findings explored the APR’s therapeutic potential for acute bacterial diarrhea, underscoring its dual role in combating oxidative stress and inflammation while modulating gut microbiota beneficially. Future investigations should explore long-term treatment effects to assess sustained benefits and broader clinical applicability.

## Figures and Tables

**Table 1:** Analysis of sequencing data for different groups.

Samples	Input	Filtered	Denoised	Merged	Non-Chimeric	Non-Singleton
CD1	249,221	217,178	215,454	179,602	172,753	172,736
CD2	86,646	80,467	79,688	77,022	73,834	73,823
CD3	112,225	105,324	103,483	94,350	64,494	64,269
CD4	72,401	55,552	54,831	51,812	51,031	51,023
CD5	83,730	68,872	67,471	57,687	52,697	52,666
ED1	64,315	59,486	58,720	55,822	54,138	54,128
ED2	250,698	212,903	210,729	177,267	176,877	176,860
ED3	78,105	73,538	72,079	64,401	42,999	42,693
ED4	126,352	107,042	105,197	92,901	80,996	80,929
ED5	130,055	115,104	114,016	110,494	110,016	110,013
TD1	75,095	70,498	69,375	62,991	45,933	45,725
TD2	80,405	75,227	73,190	61,520	41,184	40,824
TD3	76,459	72,214	70,685	64,095	42,846	42,492
TD4	135,212	115,539	112,866	97,505	86,143	86,054
TD5	62,930	54,114	53,429	51,836	50,733	50,727

**Table 2:** Analysis of alpha diversity indices.

Samples	Chao1	Faith_pd	Goods_Coverage	Observed_Species	Pielou_e	Shannon
CD1	582.0832203	958.4556284	0.996555103	476.3	0.27947093	2.485988474
CD2	255.5041792	567.6606101	0.998574081	228.1	0.279320731	2.188011412
CD3	1290.61498	95.08548944	0.994327265	1202.5	0.65048806	6.655672854
CD4	357.2362835	80.52636973	0.99972152	355	0.678150492	5.745070009
CD5	571.7104581	69.77193845	0.999022743	561.3	0.670765459	6.12584253
ED1	429.8264161	419.2213773	0.999316693	421.7	0.615342611	5.365819805
ED2	551.5114524	800.9651922	0.996910938	456.4	0.177518042	1.56818406
ED3	1182.085674	87.01808259	0.998344593	1174.2	0.71550874	7.296371476
ED4	746.050341	89.12529358	0.997117219	695	0.657047117	6.203063941
ED5	1164.977309	310.826434	0.999169718	1151.8	0.552819016	5.621986171
TD1	1009.620328	72.66430364	0.997883039	994.9	0.640729954	6.380648383
TD2	1510.369014	99.88844622	0.998630808	1506	0.724254771	7.645599344
TD3	1750.916658	87.09434071	0.996910938	1734	0.628727328	6.765034476
TD4	1020.439937	93.64806703	0.995603631	934.3	0.689977552	6.808484768
TD5	406.7303696	184.7245038	0.999623537	403.2	0.4741676	4.104083743

## Data Availability

All raw sequence data from animals were deposited in the NCBI Sequence Read Archive database under accession number PRJNA1196629 (https://dataview.ncbi.nlm.nih.gov/object/PRJNA1196629; accessed on 10 December 2024).
